# Assessment of genetic characteristics of *Aconitum* germplasms in Xinjiang Province (China) by RAPD and ISSR markers

**DOI:** 10.1080/13102818.2015.1004899

**Published:** 2015-01-29

**Authors:** Feicui Zhao, Jihong Nie, Muzhi Chen, Guirong Wu

**Affiliations:** ^a^ Department of Pharmaceutical Chemistry, Faculty of Pharmacy, Xinjiang Medical University, Urumqi, Xinjiang, PR China; ^b^ Department of Phytochemistry, Affiliated Traditional Chinese Medical Hospital, Xinjiang Medical University, Urumqi, Xinjiang, PR China

**Keywords:** *Aconitum*, genetic characteristics, RAPD, ISSR

## Abstract

*Aconitum* is a medicinal treasure trove that grows extensively on fertile pastures in Xinjiang Province (China); however, its molecular genetic characteristics are still poorly studied. We studied *Aconitum kusnezoffii* Reichb., *Aconitum soongaricum* Stapf., *Aconitum carmichaelii* Debx. and *Aconitum leucostomum* Worosch, using random amplified polymorphic DNA (RAPD) and inter-simple sequence repeat (ISSR) techniques, to evaluate their genetic relationship and potential medicinal value. Our results showed that *A.* *kusnezoffii* Reichb. and *A.* *soongaricum* Stapf*.* have close genetic relationship and cluster together. Polymorphism rates of 97.25% and 98.92% were achieved by using 15 RAPD and 15 ISSR primers, respectively. Based on Nei's gene diversity (*H*) and Shannon's index (*I*), the inter-population diversity (*H_s_*) was higher when compared with the intra-population diversity (*H_p_*). Among the three *Aconitum* populations, the coefficient of gene differentiation (*G_st_*) was 0.4358 when evaluated by RAPD and 0.5005 by ISSR. The genetic differentiation among the three *Aconitum* populations was highly significant, suggesting low gene flow (*N_m_*). This was confirmed by the estimates of gene flow (*N_m_* = 0.6473 and *N_m_* = 0.4991, based on ISSR and RAPD data, respectively). Comparing the RAPD and ISSR results, the two DNA markers proved similarly effective in the assessment of the genetic characteristics of the studied *Aconitum* populations and could be used for reliable fingerprinting and mapping in studies on *Aconitum* diversity in view of *Aconitum* suitability for development and protection.

## Introduction

Aconite is a valuable traditional Chinese herb of the family Ranunculaceae. The root of this plant is usually used for treating rheumatoid arthritis and easing of pain.[1] In recent years, it has been widely utilized in clinical therapy, e.g. lappaconitine extracted from *Aconitum sinomontanum* for inhibiting ageing and growth of cancer cells [2] or total alkaloids extracted from *Acontium flavum* Hand-Mazz for anti-inflammation and local anaesthesia.[3]

Genus *Aconitum* has 10 species and 5 varieties, and grows on fertile pastures in Xinjiang Province of China. The wild populations of this plant are a real treasure trove as a medicinal resource, but its overgrowth has become a grave threat to ranching in recent years, because aconitine is toxic to animals. To reduce the harm to ranches and at the same time enhance the medicinal utilization of aconite is a highly desired goal. In our previous work,[4] we analysed the chromatographic herbal fingerprint data and identified a variety of alkaloid monomers extracted from aconite plants growing in different regions of Xinjiang Province. By comparing the Chinese Pharmacopoeia records for *Aconitum carmichaelii* Debx. and *Aconitum kusnezoffii* Reichb. and different *Aconitum* species from Xinjiang, we found some similarities and differences in the chemical composition. Therefore, further investigation on the genetic relationship between aconite in Xinjiang and common aconite (*A.* *carmichaelii* Debx. and *A.* *kusnezoffii* Reichb.) would be important for developing its potential medicinal value.

Molecular marker techniques are powerful and valuable tools used in the analysis of medicinal plants. Among the markers, RAPD (random amplified polymorphic DNA) and ISSR (inter-simple sequence repeat) are generally preferred because of their sensitivity, simplicity and cost-effectiveness. Both RAPD and ISSR markers have been successfully applied to detect genetic similarities or differences in various herbs.[5] As these two types of markers amplify different regions of the genome, when applied together, they allow better analysis of genetic identity and variation.

The aim of the present study was to assess the genetic identity between representatives of genus *Aconitum* in Xinjiang and *A.* *carmichaelii* Debx. and *A.* *kusnezoffii* Reichb., by RAPD and ISSR markers. The obtained information about the genetic characteristics will be valuable for screening a variety of Xinjiang representatives of genus *Aconitum*.

## Materials and methods

### Sample collection and DNA extraction

Fifteen aconite plants belonging to four species were chosen: *Aconitum leucostomum* Worosch., *Aconitum soongaricum* Stapf., *A.* *carmichaelii* Debx. and *A.* *kusnezoffii* Reichb. The details of the accessions and their geographic origin are listed in Table 1. The roots of plants were independently harvested, frozen in liquid nitrogen and stored at −80 °C until DNA extraction. DNA was extracted from 100 mg of root material, using a modified Doyle method.[6] Finally, the extracted DNA samples were quantified with a spectrophotometer (Nanadrop 2000, Thermo Scientific) and diluted to 50 ng/μL in Tris-EDTA buffer; then they were stored at −80 °C for further analyses.

**Table 1.** *Aconitum* specimens and their geographic origin.

No.	Species	Geographic location
1	*Aconitum kusnezoffii* Rdichb.	Sichuan Province, China
2	*Aconitum carmichaelii* Debx.	Sichuan Province, China
3	*Aconitum soongaricum* Stapf.	Tokkuztara County, Xinjiang Province, China
4	*Aconitum leucostomum* Worosch	Qinggil County, Xinjiang Province, China
5	*Aconitum soongaricum* Stapf.	Nilka County, Xinjiang Province, China
6	*Aconitum soongaricum* Stapf.	Nilka County, Xinjiang Province, China
7	*Aconitum leucostomum* Worosch	Burqin County, Xinjiang Province, China
8	*Aconitum leucostomum* Worosch.	Habahe County, Xinjiang Province, China
9	*Aconitum leucostomum* Worosch	Burqin County, Xinjiang Province, China
10	*Aconitum leucostomum* Worosch.	Fuyun County, Xinjiang Province, China
11	*Aconitum leucostomum* Worosch	Nilka County, Xinjiang Province, China
12	*Aconitum carmichaelii* Debx.	Sichuan Province, China
13	*Aconitum carmichaelii* Debx.	Sichuan Province, China
14	*Aconitum kusnezoffii* Rdichb.	Sichuan Province, China
15	*Aconitum leucostomum* Worosch	Nilka County, Xinjiang Province, China

### RAPD analysis

For polymerase chain reaction (PCR), 20 ng of genomic DNA were amplified in a volume of 50 μL containing 10X PCR buffer (10 mmol/L of Tris-HCl, pH 8.3; 50 mmol/L of KCl_2_), 2.5 mmol/L of MgCl_2_, 0.2 mmol/L of each deoxyribonucleoside triphosphate (dNTP), 0.4 μmol/L primer and 2 U of *Taq* DNA polymerase, by means of a thermal cycler (MJ-Mini, BioRad, USA). The cycling programme began with an initial 7 min at 95 °C, followed by 45 cycles at 95 °C for 45 s, 34 °C for 30 s and 72 °C for 90 s, plus a final 10 min at 72 °C and storage at 4 °C. Amplification products were separated by electrophoresis in 1.5% agarose gels. A total of 120 single primers (ShengGong Biotechnology Inc, CHN) were used in the PCR programme, and as a result, 15 primers that amplified polymorphic bands were selected. The sequences of the 15 primers are shown in Table 2.

**Table 2.** Primers sequences.

RAPD primers sequence			ISSR primers sequence		
Primer	RAPD primer sequence (5′–3′)	Polymorphic rate (%)	Primer	ISSR primer sequence (5′--3′)	Polymorphic rate (%)
S14	TCCGCTCTGG	100	UBC814	(CT)8A	100
S28	GTGACGTAGG	100	UBC815	(CT)8G	100
S31	CAATCGCCGT	90.91	UBC818	(CA)8A	100
S36	AGCCAGCGAA	100	UBC822	(TC)8A	100
S38	AGGTGACCGT	100	UBC823	(TC)8C	100
S39	CAAACGTCGG	90.91	UBC824	(TC)8G	100
S40	GTTGCGATCC	88.89	UBC835	(AG)8YC	100
S41	ACCGCGAAGG	100	UBC844	(CT)8RC	100
S52	CACCGTATCC	100	UBC845	(CT)8RG	100
S54	CTTCCCCAAG	100	UBC846	(CA)8RT	100
S79	GTTGCCAGCC	100	UBC852	(TC)8RA	100
S81	CTACGGAGGA	90.00	UBC869	(GTT)6	88.89
S90	AGGGCCGTCT	100	UBC876	(GATA)2(GACA)2	100
S106	ACGCATCGCA	93.33	UBC879	(CTTCA)3	88.89
S112	ACGCGCATGT	100	UBC895	AGAGTTGGTAGCTCTTGATC	100

### ISSR analysis

ISSR reactions were performed in a volume of 50 μL containing 25 ng of template DNA, 10X PCR buffer (10 mmol/L of Tris HCl, pH 8.3; 50 mmol/L of KCl_2_), 2.5 mmol/L of MgCl_2_, 0.2 mmol/L of each dNTP, 0.4 μmol/L primer and 2 U of *Taq* DNA polymerase, by means of a thermal cycler (MJ-Mini, BioRad, USA). PCR amplification was performed as follows: initial denaturation at 95 °C for 7 min, followed by 45 cycles at 95 °C for 30 s, 48 °C–60 °C for 45 s and 72 °C for 90 s and a final 10 min extension at 72 °C. Amplification products were separated by electrophoresis in 1.5% agarose gels. A total of 100 primers (ShengGong Biotechnology Inc, CHN) were used in the PCR programme, and as a result, 15 primers that amplified polymorphic bands were selected. The sequences of the 15 primers are shown in Table 2.

### Data analysis

For the two types of molecular markers, several independent samples from each *Aconitum* species collected from several locations were tested, and only clear, unambiguous and reproducible bands amplified in both cases were considered. The numbers of polymorphic and monomorphic amplification products were determined for each primer for 15 individuals. To avoid taxonomic weighing, the intensity of bands was not taken into consideration, and only the presence of a band was taken as an indicative. The basic parameters for genetic diversity were calculated with the POPGENE application.[7] The polymorphism of amplification products (*P*), the number of observed alleles (*N_a_*), the mean number of effective alleles (*N_e_*), mean Nei's gene diversity index (*H*), the Shannon index (*I*) and the level of gene flow (*N_m_*) [8,9] were determined. Inter-populations diversity (*H_s_*), total gene diversity (*H_t_*) and Nei's coefficient of gene differentiation (*G_st_*) [10] were calculated using the POPGENE 32 software: *G_st_* = (1 − *H_s_*/*H_t_*); *N_m_,* estimate of gene flow from *G_st_*, *N_m_* = 0.5 × (1° *G*_st_)/*G*_st_ *_._*. The level of similarity among individuals was established as a percentage of polymorphic bands, and a matrix of genetic similarity was compiled using Dice's coefficient.[11] Dendrograms of the genetic relationship among the 15 individuals of *Aconitum* were generated by applying the unweighted pair-group arithmetic average (UPGMA) method.[12]

## Results and discussion

### *RAPD and ISSR fingerprinting*

**Figure 1.**
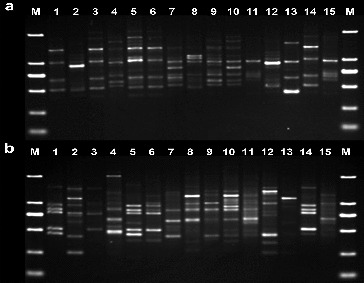
RAPD electrophoretic analysis of 15 *Aconitum* specimens (**a**: S112 primer; **b**: S41 primer). M: DL2000 DNA marker: 100 bp, 250 bp, 500 bp, 750 bp, 1000 bp, 2000 bp.

**Figure 2.**
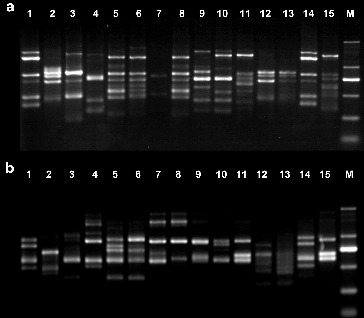
ISSR electrophoretic analysis of 15 *Aconitum* specimens (**a**: UBC881 primer; **b**: UBC823 primer). M: DL2000 DNA marker: 100 bp, 250 bp, 500 bp, 750 bp, 1000 bp, 2000 bp.

Following screening, 15 primers were chosen for the evaluation of genetic diversity in the four *Aconitum* species by RAPD and ISSR. The other primers did not generate any amplification products or stable bands. The results from the amplification obtained using the 15 selected primers, the total number of amplification products and the total number of polymorphic fragments, are summarized in Table 3. For the 15 *Aconitum* specimens analysed, a total of 182 bands and a polymorphism index (*P*) of 97.25% were obtained by RAPD, and a total of 185 bands and a polymorphism index (*P*) of 99.02%, by ISSR (Table 3). The number of bands per primer ranged from 7 to 18, with an average of 12 bands per primer and fragment size ranging from 0.1 to 2 kb in RAPD and ISSR (Figures 1 and 2).

**Table 3.** Degree of polymorphism for RAPD and ISSR primers in 15 *Aconitum* specimens.

Marker	Number of primers	Total bands	Mean bands per primer	Range of bands (bp)	Total polymorphic bands	Polymorphic bands rate (%)
RAPD	15	182	12.13	100--2000	177	97.25
ISSR	15	185	12.33	100--2000	183	98.92

### *RAPD and ISSR clustering analysis*

**Figure 3.**
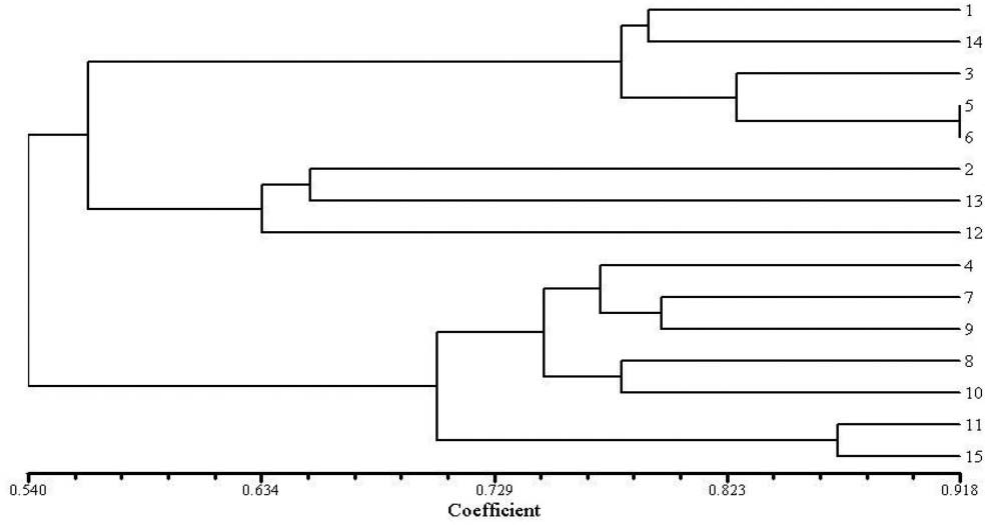
Dendrogram plot of 15 *Aconitum* specimens by UPGMA cluster analysis (RAPD).

**Figure 4.**
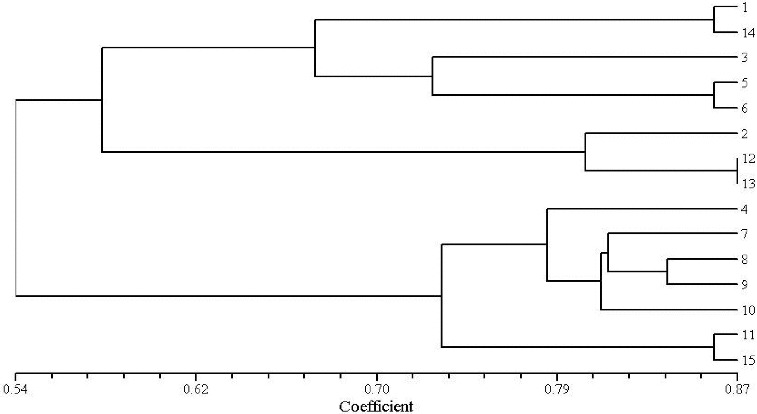
Dendrogram plot of 15 *Aconitum* specimens by UPGMA cluster analysis (ISSR).

To evaluate the effectiveness of RAPD and ISSR markers for the study of genetic relationships in genus *Aconitum*, the genetic similarity coefficient was calculated. Cluster analysis was performed for the results obtained by the 15 RAPD and 15 ISSR primers identified to be polymorphic in the 15 studied aconite specimens. UPGMA dendrograms were constructed using SHAN neighbour-joining tree (Figures 3 and 4). The similarity coefficient of the genetic relationships among the 15 *Aconitum* specimens belonging to three populations was determined to be 0.62 based on the RAPD and ISSR data.

The obtained results revealed that *A.* *kusnezoffii* Reichb. and *A.* *soongaricum* Stapf. had close genetic relationship and were clustered together. Then, they were clustered with *A.* *carmichaelii* Debx. Unlike them, *A.* *leucostomum* Worosch. was a unique species, distinct from the other studied *Aconitum* species with a similarity coefficient of 0.54. RAPD and ISSR clustering analysis (Figures 3 and 4) showed that both techniques have good consistency and repeatability.

### *Genetic diversity and genetic differentiation*

Based on genetic identity, the 15 *Aconitum* specimens were divided into three clusters in the clustering analysis: P1: *A.* *kusnezoffii* Reichb. and *A.* *soongaricum* Stapf.; P2: *A.* *carmichaelii* Debx.; P3: *A.* *leucostomum* Worosch. At the population level, the average values of *N_a_*, *N_e_*, *H* and the percentage of polymorphism were 1.9725% and 1.9892%, 1.5130% and 1.4948%, 0.3072% and 0.3021%, 51.10% and 45.41%, using two molecular markers (RAPD and ISSR), respectively (Table 4). The estimates of mean Shannon's index (*I*) values for the three populations, based on RAPD and ISSR, were similar: 0.4686 and 0.4654, respectively. These high *I* values at the population level indicate that the populations belong to different species.

**Table 4.** Mean genetic data of three *Aconitum* specimens based on RAPD and ISSR markers.

Markers	population	*N_a_*	*N_e_*	*H*	*I*	*p*	*POL%*
RAPD	P1	1.3956 ± 0.4903	1.2513 ± 0.3649	0.1447 ± 0.1958	0.2151 ± 0.2815	72	39.56
	P2	1.5385 ± 0.4999	1.3401 ± 0.3676	0.2007 ± 0.1982	0.2994 ± 0.2874	98	53.85
	P3	1.5989 ± 0.4915	1.3018 ± 0.3428	0.1845 ± 0.1838	0.2647 ± 0.2644	109	59.89
	Mean	1.9725 ± 0.1639	1.5130 ± 0.3243	0.3072 ± 0.1515	0.4686 ± 0.1911	93	51.10
ISSR	P1	1.5459 ± 0.4992	1.2996 ± 0.3433	0.1818 ± 0.1881	0.2770 ± 0.2731	101	54.59
	P2	1.2649 ± 0.4425	1.1502 ± 0.2781	0.0926 ± 0.1605	0.1406 ± 0.2395	49	26.49
	P3	1.5514 ± 0.4987	1.2979 ± 0.3531	0.1785 ± 0.1911	0.2718 ± 0.2745	102	55.14
	Mean	1.9892 ± 0.1037	1.4948 ± 0.3098	0.3021 ± 0.1422	0.4654 ± 0.1757	84	45.41

Note: *N_a_* – observed number of alleles; *N_e_* *–* effective number of alleles; *H* – Nei's gene diversity; *I* – Shannon's Information index; *p* – number of polymorphic loci; *POL* – percentage of polymorphic loci

The average coefficient of genetic differentiation (*G_st_*) was 0.4358 and 0.5005, respectively, for RAPD and ISSR, among the three *Aconitum* populations (Table 5). This high genetic differentiation among the three *Aconitum* populations based on both RAPD and ISSR data, suggested a low level of gene flow. This differentiation was in line with the estimates of gene flow (*N_m_* = 0.6473 and *N_m_* = 0.4991, based on ISSR and RAPD data, respectively).

**Table 5.** Genetic populations structure and estimate of gene flow within the populations of *Aconitum.*

Markers	*H_s_*	*H_t_*	*G_st_*	*N_m_*
ISSR	0.1766 ± 0.0123	0.3130 ± 0.0218	0.4358	0.6473
RAPD	0.1510 ± 0.0087	0.3022 ± 0.0203	0.5005	0.4991

Note: *H_s_* – inter-populations diversity; *H_t_* *–* total variability; *G_st_*, Inter-populations differentiation, *G_st_* = (1 − *H_s_*/*H_t_*); *N_m_,* estimate of gene flow from *G_st_*, *N_m_ =* 0.5 × (1 − *G_st_*)/*G_st_*.

These results are interesting, as cultivated *Aconitum* *carmichaeli* Debx, and more precisely its lateral roots, is used in one of the traditional Chinese medicine herbs, *Radix Aconiti Lateralis Preparata*. Due to their wide range of clinical applications, aconite plants have become an important medicine resource. *Aconitum kusnezoffii* Reichb., *A.* *soongaricum* Stapf., *A.* *leucostomum* Worosch., *Aconitum karakolicum* Rapaics and *Aconitum leucostomum* var. *nalatiensis* are widespread in several regions in Xinjiang Province.[13] Such medicinal materials are treated as a substitute for the use of *A.* *carmichaelii* and *A.* *kusnezoffii*, and are used frequently in traditional medicine in the Northwest Territories. However, their quality and purity must meet the market requirements. Aconite products have complex sources, with high-quality medicinal material sometimes mixed together with inferior varieties. It is difficult to classify and genetically analyse them, using traditional morphological characteristics and pedigree analysis. Both RAPD and ISSR technology are valid and useful tools for classification and genetic analysis and have been successfully used for the identification of *Aconitum* species.[14] These methods can detect a higher level of polymorphism than morphological analysis.

Both RAPD and ISSR are effective techniques for assessment of the genetic diversity of *Aconitum* spp. medicinal material. They can effectively reveal the genetic relationship between medicinal plant materials. In this way, inter- and intra-species DNA variability can be analysed at the molecular level, which is particularly important in breeding and cultivation of new varieties.

## Conclusions

This study is, to the best of our knowledge, the first report on the genetic characteristics of such a range of aconite germplasm in Xinjiang Province. The obtained RAPD and ISSR data indicated high level of genetic differentiation and low gene flow among the studied species. *Aconitum leucostomum* Worosch. was clustered separately, suggesting that it could be considered as a relatively independent Xinjiang aconite species, because it has farther genetic distance than other species. That is why it may be a promising target for future investigations into its medicinal value. The obtained results are also relevant in view of the suitability of the used sets of RAPD and ISSR primers for development and protection of traditional Chinese medicinal material, and regulation of the Chinese medicinal material market.

## References

[cit0001] Anderson JA, Churchill GA, Autrique JE, Tanksley SD, Sorrells ME (1993). Optimizing parental selection for genetic linkage maps. Genome.

[cit0002] Archak S, Ambika B, Gaikwad D, Gautam EV, Rao VB, Swamy KRM, Karihaloo JL (2003). DNA fingerprinting of India cashew (*Anacardium occidentale* L.) varieties using RAPD and ISSR techniques. Euphytica.

[cit0003] Brijesh S, Daswani PG, Tetali P (2006). Studies on *Pongamia pinnate* (L.) Pierre leaves: understanding the mechanisms of action in infectious diarrhea. J Zhejiang Univ Sci B.

[cit0004] Liu SM, Nie JH, Pan R, Zhao FC (2012). Determination of the content of total alkaloid in Xinjiang genus Aconitum. J Xinjiang Med Univ.

[cit0005] Bussell JD (1999). The distribution of random amplified polymorphic DNA (RAPD) diversity among populations of *Isotoma petraea* (Lobeliaceae). Mol Ecol.

[cit0006] Doyle JJ, Doyle JL (1987). A rapid DNA isolation procedure for small quantities of fresh leaf tissue. Phytochem Bull.

[cit0007] Ferrante M, Yeh JTC (2010). Head and flux variability in heterogeneous unsaturated soils under transient flow conditions. Water Resour Res.

[cit0008] Slatkin M (1985). Gene flow in natural populations. Annu Rev Ecol S.

[cit0009] Mcdermott JM, Mcdonald BA (1993). Gene flow in plant pathosystems. Annu Rev Phytopathol.

[cit0010] Nei M (1973). Analysis of gene diversity in subdivided populations. PNAS.

[cit0011] Dice LR (1945). Measures of the amount of ecological association between species. Ecology.

[cit0012] Fu YB, Ferdinandez YSN, Phan AT, Coulman B, Richards KW. AFLP variation in four blue grama seed sources. Crop Sci. 2004;44(1):283–288.

[cit0013] Zhang F, Liu Y, Cai DM (2012). A new variety of *Aconitum* L. from Xinjiang, China. Northwest Pharm J.

[cit0014] Luo Q, Ma DW, Wang YH (2006). ISSR identification of genetic diversity in *Aconitum carmichaeli*. Chinese Traditional and Herbal Drugs.

